# Microstructural Mechanisms of Concrete Degradation Under Different Coal Gangue Sand Replacement Ratios

**DOI:** 10.3390/ma18204787

**Published:** 2025-10-20

**Authors:** Yukai Cai, Wenhua Zha, Tao Xu, Chao Ji, Yaozong Li

**Affiliations:** 1 School of Civil and Architectural Engineering, East China University of Technology, Nanchang 330013, China; 18702515606@163.com (Y.C.);; 2 Surveying and Mapping Brigade, Geological Bureau of Jiangxi Province, Nanchang 330001, China

**Keywords:** coal gangue manufactured sand, concrete performance degradation, crack propagation, interfacial transition zone (ITZ)

## Abstract

Coal gangue manufactured sand (CGS), a sustainable substitute for natural sand, offers both resource and environmental benefits; however, the micro-mechanisms underlying performance deterioration at different replacement levels remain unclear. In this study, cube specimens with 25%, 50%, 75%, and 100% CGS were tested in uniaxial compression, and the results were integrated with PFC2D discrete-element simulations and SEM observations to establish an energy–force-chain–crack coupling framework. Experiments and simulations showed close agreement in peak stress, peak strain, and overall curve shape (errors generally <5%). With increasing replacement, the interfacial transition zone (ITZ) evolves from a dense three-phase ITZ (NS–CGS–CA; natural sand–CGS–coarse aggregate) to a degraded two-phase ITZ (CGS–CA), accompanied by more pores and microcracks; the proportion of Adhesive cracks decreases while Cohesive (intra-particle) cracks increase. Concurrently, continuous force-chain networks deteriorate into localized short-chain clusters; the peak and fraction of strain-energy decrease, whereas frictional/damping dissipation rises—together driving a macroscopic transition from ductile to brittle behavior. At 28 d, SEM images and DEM evolution of cracks/force chains/energy exhibit strong consistency, further confirming that low replacement (25% and 50%) favors stable load-transfer paths and suppresses early cracking, whereas high replacement (75% and 100%)—through ITZ degradation and force-chain instability—induces more concentrated cracking and higher energy dissipation, thereby diminishing mechanical performance.

## 1. Introduction

With the rapid development of the global construction industry, particularly in China, the excessive exploitation of natural sand (NS) and gravel resources has triggered a series of environmental and economic issues, including ecosystem degradation, soil erosion, and rising construction material costs. To alleviate the shortage of sand resources and promote the recycling of solid waste, the development of green building materials capable of replacing natural aggregates has become a major research focus in civil engineering and materials science [1,2,3]. Among various solid wastes, coal gangue has gradually become a priority for resource utilization due to its large output and the significant risks associated with stockpiling.

As a major by-product of coal mining, coal gangue accounts for roughly 10–20% of raw coal output in China, with an annual generation of 150–200 million tons and historical stockpiles exceeding 10 billion tons [4,5]. Prolonged stockpiling occupies land and entails environmental risks such as spontaneous combustion, slope failure, and water contamination [6]. With advances in processing, coal gangue can be crushed and sieved into manufactured sand—Coal Gangue Sand (CGS)—to partially replace NS in concrete production. This approach both alleviates the depletion of NS and aligns with national “Dual-Carbon” goals, contributing to energy conservation, emission reduction, and ecological protection [7]. Consequently, the resource utilization of coal gangue meets the practical needs of waste management and supports the green transition of the construction sector, making CGS concrete an emerging hotspot in sustainable materials research.

Despite these advantages, the use of coal gangue as concrete aggregate is constrained by mechanical and durability limitations. When used as coarse aggregate (CA), compressive strength generally decreases with increasing replacement ratio, with pronounced degradation beyond about 45% [8,9,10]. The primary cause is the relatively low strength and high crushing index of coal gangue, which induces a “weak-inclusion” effect: particles fracture prematurely under load, undermining overall capacity [11,12]. As a fine aggregate, the recommended replacement is also limited—most studies suggest not exceeding ~20%. At moderate replacement levels, however, reactive components in coal gangue (e.g., SiO_2_, Al_2_O_3_) can interact with cement hydration products to form additional C–S–H and AFt, densifying the interfacial transition zone (ITZ) and contributing to early-age strength [13,14]. At higher replacement ratios, the inherently higher porosity and water absorption of gangue particles deteriorate performance, including reduced strength and increased drying shrinkage [15,16]. Durability studies have further examined freeze–thaw resistance, sulfate attack, and carbonation [17,18]. Relative to natural-sand concrete, CGS concrete typically exhibits higher porosity and more pronounced ITZ defects, which impair long-term serviceability [19,20]. To mitigate these shortcomings, strategies such as supplementary cementitious materials and particle surface modification have been proposed to enhance overall performance and expand engineering applicability [21,22].

However, much of the existing work remains focused on macroscopic properties, with limited attention to the micro-mechanisms underlying performance degradation—particularly the progressive deterioration of the ITZ and its impact on meso-mechanical behavior. As the weakest region between aggregates and paste, the ITZ is critical for crack initiation and propagation [23,24,25]; its degradation disrupts continuous stress-transfer paths, weakens force-chain stability, and shifts the balance between energy storage and dissipation during loading. The discrete element method (DEM) can explicitly model grain-scale interactions and is therefore well suited for meso-scale mechanism studies [26,27]. PFC2D-based simulations have reproduced macroscopic responses in materials such as recycled aggregate concrete and cemented soils, supporting their validity [28,29,30,31,32,33]. Nevertheless, DEM studies on CGS concrete or the ITZ often address isolated aspects—for example, the influence of ITZ properties on damage accumulation and cracking [19], visualization of force-chain transmission and reorganization under load [17], or energy absorption/dissipation under dynamic impact [32]. A key gap remains: a unified numerical framework that systematically reveals how CGS replacement ratio governs the fully coupled behavior of “force-chain evolution–crack propagation–energy transformation,” together with direct microstructural corroboration.

To fill this gap, this study integrates uniaxial compression tests, PFC2D simulations, and scanning electron microscopy (SEM) to investigate the micro-mechanisms of performance degradation in CGS concrete at different replacement ratios (25%, 50%, 75%, 100%). The main contributions are:

(1) Establishing a unified energy–force-chain–crack coupling framework to evaluate the global, synergistic evolution of meso-scale parameters with replacement ratio;
(2) Elucidating the intrinsic link between the structural stability of the force-chain network and internal energy reallocation—specifically, the shift from strain-energy-dominated storage to frictional/damping-dominated dissipation—as the driver of macroscopic brittle transition;
(3) Providing direct experimental support via SEM for numerically revealed ITZ mechanisms (e.g., transformation from a dense “three-phase” to a porous “two-phase” structure), achieving cross-scale correspondence between micro-morphology and meso-mechanics.

Beyond environmental benefits, this work offers technical value: the PFC2D platform enables explicit tracking of force-chain evolution and progressive particle degradation within the ITZ under uniaxial loading, offering visual insight into meso-scale degradation at varying replacement levels. The proposed energy–force-chain–crack framework supplies a fresh analytical lens for understanding performance deterioration in CGS concrete and provides a theoretical basis and technical guidance for determining optimal CGS content, improving ITZ quality, and advancing the engineering application of green concrete.

## 2. Materials and Methods

### 2.1. Macroscopic Experimental Program

#### 2.1.1. Preparation of Concrete with Coal Gangue Manufactured Sand

**Figure 1:**
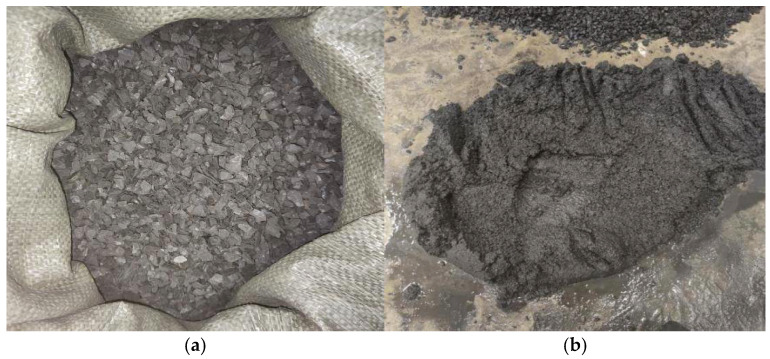
Coarse and fine aggregates of coal gangue. (**a**) Coal gangue (10–16 mm); (**b**) machined coal gangue sand (0–4.75 mm).

Ordinary Portland cement (P.O 42.5) was used as the binder. The fine aggregate consisted of a blend of NS manufactured CGS. The NS was sourced from the Ganjiang River Sand Group (Nanchang, China), while the coal gangue was obtained from the Qujiang Coal Mine (Fengcheng, China) and processed into CGS by crushing and sieving (Figure 1). Sieve analysis showed a fineness modulus of 2.74, corresponding to medium sand; the grading met the Zone II requirements of GB/T 14684-2022 [34] (Sand for Construction).

**Figure 2:**
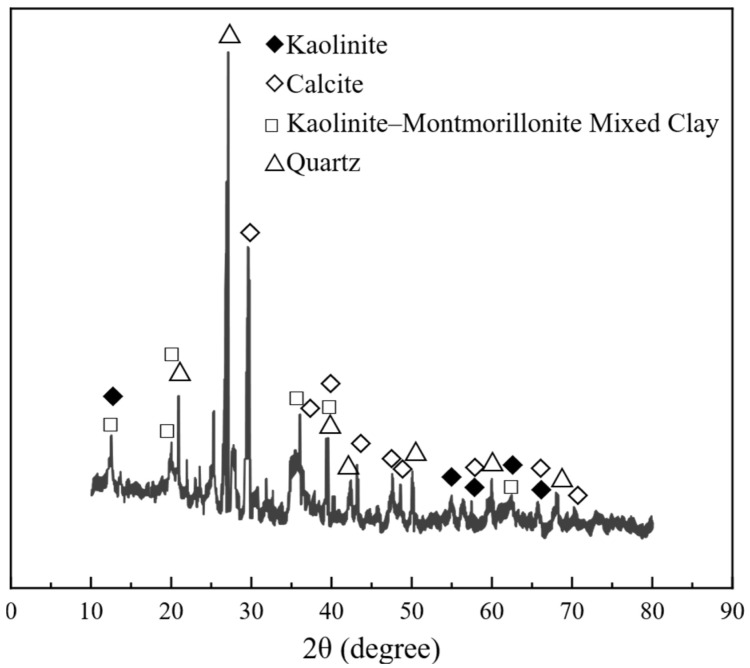
XRD pattern of coal gangue sand.

The key physical properties of CGS are listed in Table 1, and its mineral composition is shown in Figure 2. Tests for bulk/apparent density, void ratio, and soundness (or crushing-related indices) followed GB/T 14684-2022 and related national standards. In all mixtures, the CA was natural crushed stone, while the fine aggregate was a blend of river sand and CGS at different proportions. To evaluate the effect of CGS content, four mixes (J1–J4) were designed with CGS mass-replacement ratios of 25%, 50%, 75%, and 100%, and uniaxial compression tests were conducted; detailed mix proportions are provided in Table 2.

This study selects 25% as the initial replacement ratio for CGS, primarily based on research objectives and literature evidence:

(1) Research Objective: The aim is to initiate observations near the identifiable threshold of mechanical response to obtain mesoscopic mechanical evidence with sufficient resolution. According to existing research (e.g., M. R. Lavanya et al. [35]), the influence of alternative aggregates typically begins to exhibit stable and reproducible patterns only after the replacement ratio reaches approximately 25%. At lower ratios, the mechanical response is often too weak to reveal clear mechanisms, due to the difficulty in forming effective force chain skeletons and interfacial interactions.

(2) Literature Evidence: Multiple studies indicate the existence of a critical threshold range for the replacement ratio, which affects both macroscopic strength and ITZ structure. For instance, Yu et al. [25], investigating a replacement gradient from 0% to 100%, found that the mechanical properties at a 25% replacement ratio were closest to those of the reference group, making it an ideal benchmark for comparison. Jia et al. [36] further specified that when the replacement ratio is below 20%, the strength reduction is insignificant (approximately 95% of the reference group), whereas beyond 30%, strength deterioration accelerates markedly.

In summary, this study establishes 25% as the initial replacement ratio and sets increasing gradients of 50%, 75%, and 100% based on this point, thereby systematically revealing the micro-macro response mechanisms as the replacement ratio increases.

#### 2.1.2. Curing of Specimens

**Figure 3:**
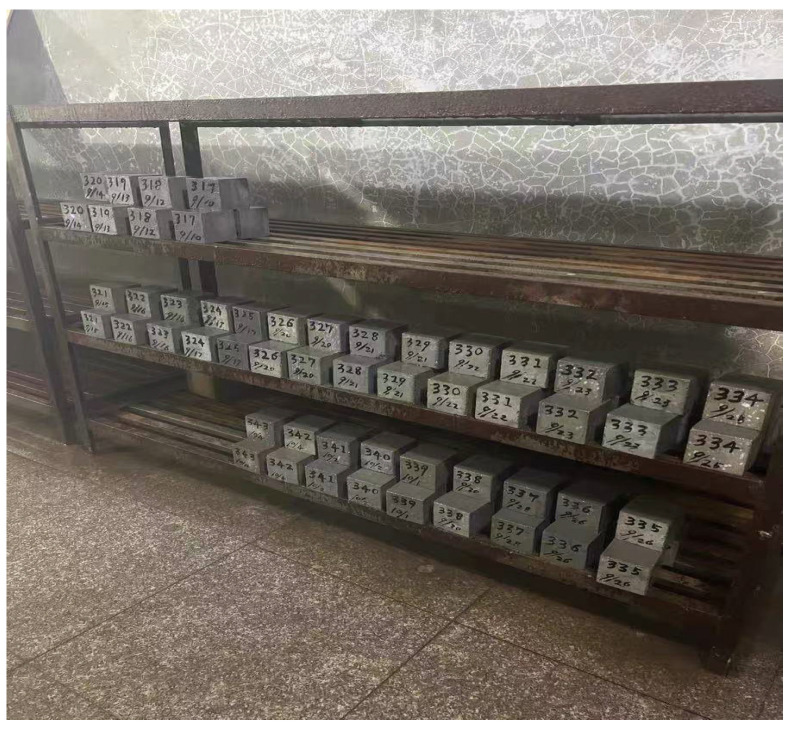
Curing of specimens.

Cubic specimens (100 × 100 × 100 mm) were demolded after 24 ± 2 h and cured under standard conditions (20 ± 2 °C, RH ≥ 95%). Curing ages were 1, 3, 7, and 28 d (Figure 3). Geometric tolerances were controlled within ±1.0 mm; end-face flatness and parallelism conformed to GB/T 50081-2019 [37] (Standard for Test Methods of Mechanical Properties of Ordinary Concrete). Before testing, free surface water was removed by wiping, and specimens were equilibrated at 20 ± 2 °C for ≥2 h to ensure thermal–hygrometric stability; tests were then conducted within the scheduled time window. Curing and testing procedures complied with GB/T 14684-2022 [34] (Sand for Construction) and GB/T 50081-2019, respectively.

#### 2.1.3. Compressive Strength Test on Cubes

**Figure 4:**
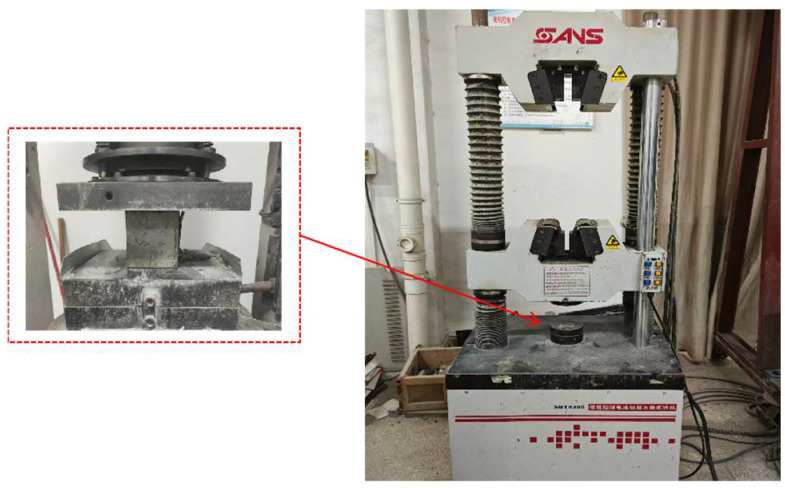
Schematic of the laboratory uniaxial compression test setup.

Compressive tests were performed on a servo-controlled universal testing machine under displacement control at a loading rate of 0.01 mm·s^−1^. To mitigate end restraint and friction, steel platens with a low-friction interlayer were used, and a small preload was applied to eliminate seating gaps prior to the formal loading. Load–displacement data were continuously recorded throughout the tests (Figure 4).

The nominal stress was computed as $\sigma_{max}=F/A_{0}$ (where F is the axial load and A_0_ is the initial loaded area), and the nominal strain as $\varepsilon =∆H/H_{0}$ (where ∆H is the axial displacement and H0 is the initial height). The peak stress $\sigma_{max}$ and the corresponding peak strain $\varepsilon_{peak}$ were identified directly from the stress–strain curves. For each mix and curing age, the number of replicates was n = 3.

Results are reported using a unified statistical convention: mean ± standard deviation (SD) together with the 95% confidence interval (CI), computed as:

$$\mathrm{Mean}\;=\;{\bar{x},SD=s,CI}_{95\%}=\bar{x}\pm t_{0.975,n-1}\times \frac{s}{\sqrt{n}}$$

where $\bar{x}$ is the sample mean, s is the sample standard deviation, and $t_{0.975,n-1}$ is the two-tailed critical value of the t distribution with *n* − 1 degrees of freedom (for *n* $=3,t_{0.975,2}\approx 4.303$). In the tables, symbols/lines denote means and error bars denote 95% CIs (*n* = 3). For CGS concrete at different curing ages (1, 3, 7, and 28 days), the standard deviations and 95% confidence intervals of the peak stress and peak strain, after processing and calculation, are presented in Table 3 and Table 4.

Although mechanical and strain data were obtained at 1, 3, 7, and 28 d, subsequent DEM-experiment comparisons focus on the 28 d specimens for two reasons consistent with common practice in this field: (1) 28 d is the most comparable reference point in relevant codes/standards and the literature [25,38,39,40], representing near-complete hydration and a mature strength/ITZ state; (2) focusing on 28 d minimizes confounding from early-age shrinkage, moisture fluctuations, and interfacial instability, thereby improving the representativeness and robustness of the DEM-experiment comparison.

### 2.2. Two-Dimensional DEM Modeling and Parameter Calibration of CGS Concrete

#### 2.2.1. Construction of the 2D DEM Model

To simulate the meso-scale behavior of concrete incorporating CGS, a two-dimensional random aggregate model was built in PFC2D. Concrete was represented as a two-phase particulate system comprising CA and mortar: the CA was natural crushed stone with a particle-size range of 10–16 mm, while the mortar matrix consisted of NS and CGS (NS: 2.5–5.0 mm; CGS: 3.25–4.75 mm). In the 2D domain, the target area fractions of CA and mortar were set to 70% and 30%, respectively.

To capture the effect of CGS incorporation, CGS partially replaced NS in the mortar at different levels. By adjusting the proportion of CGS to NS, the influence of the replacement level on the meso-mechanical response was investigated numerically. Within the mortar phase, NS and CGS were treated as two particle groups under the same parallel-bond (PB) framework. The replacement ratio ρ was implemented by relabeling approximately ρ% of the mortar-sand particles as CGS within each size bin, while keeping the global particle-size distribution (PSD), total particle count, and target porosity unchanged to avoid PSD bias.

**Figure 5:**
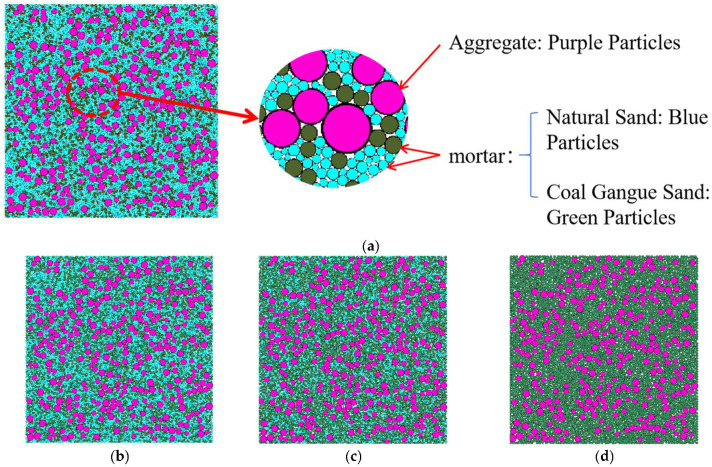
Random aggregate models of specimens with different mix proportions. (**a**) 25% replacement rate of CGS, (**b**) 50% replacement rate of CGS, (**c**) 75% replacement rate of CGS, (**d**) 100% replacement rate of CGS.

The computational domain was a 100 mm × 100 mm square. Circular particles were randomly generated in PFC2D with an initial uniform porosity of 0.08. Example random-aggregate models at different replacement ratios are shown in Figure 5 (purple: CA; blue: NS; green: CGS). To ensure a reasonable PSD and adequate statistical representativeness, each model used no fewer than 10,000 particles.

#### 2.2.2. PFC2D-Based Discrete Element Simulation

**Figure 6:**
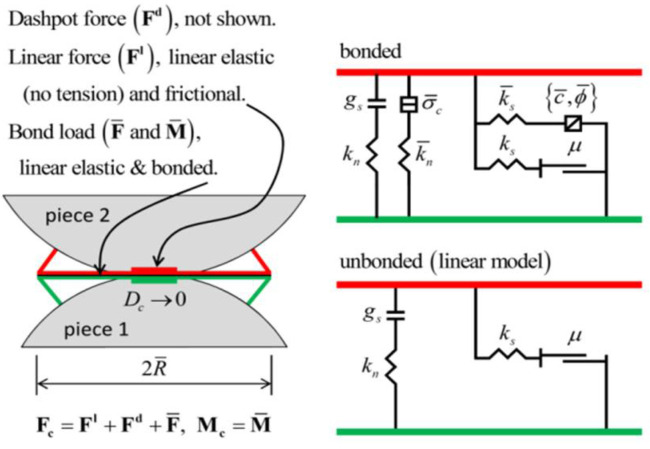
Schematic diagram of the parallel bond (PB) mechanical model.

**Figure 7:**
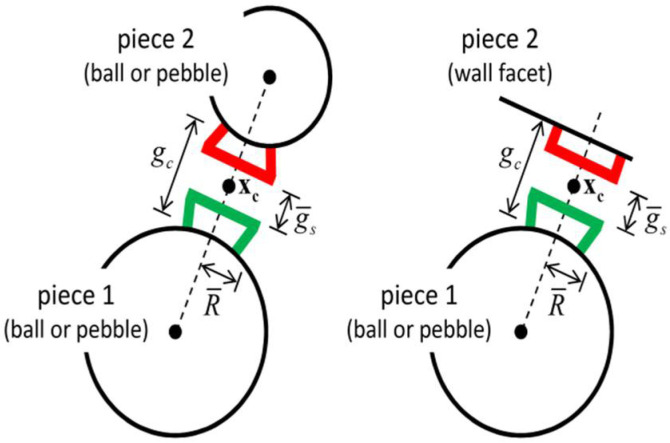
Schematic diagram of the parallel bond (PB) model.

The linear parallel-bond (PB) model was adopted to describe micromechanical interactions between particles. In addition to normal and shear contact forces, the PB formulation incorporates moment transmission and bond-strength criteria, thereby capturing interfacial bonding, progressive damage, and bond failure during loading (Figure 6 and Figure 7).

(1) Updates of contact force and moment

Within the PB framework, the contact force and contact moment are updated by linear-contact and bond contributions:

$$F_{c}=F_{l}+F_{d}+F_{b},\;M_{C}=M_{b},$$

where *Fc* and *Mc* are the resultant contact force and moment; *F_d_* denotes damping, *F_l_* the linear-contact (inertial) term, and *F_b_* and *M_b_* the parallel-bond force and moment, respectively. The bonded force is decomposed into normal and shear parts:

$$F_{b}=F_{n}n+F_{s}t,$$

with linear relations to relative displacements:

$$F_{n}=k_{n}\delta_{n},F_{s}=k_{s}\delta_{s}$$

where *n* and *t* are the unit normal and tangential vectors, *δn* and *δs* are the accumulated normal and tangential relative displacements, and *k_n_* and *k_s_* are the normal and shear stiffnesses. The bond moment M_b_ accounts for bending between bonded particles.

(2) Bond formation and failure criterion

An elastic interface (parallel bond) is created once the normal approach satisfies the bonding condition; forces and moments start from zero and accumulate with relative displacement. When a normal or shear strength limit is exceeded, the bond breaks, and the contact switches from “bonded” to “unbonded.” In the original convention, bond formation is judged by the normal overlap g_c_:

$$g_{c}>0\to bonded$$

while under tangential sliding the bond remains intact unless a strength threshold is reached. The cumulative tangential displacement is tracked by incremental summation:

g_s_ = ∑Δδ_s_

where Δδs is the tangential displacement increment.

(3) Parameters and applicability

The micro-parameters of the PFC model were determined primarily by calibrating against laboratory uniaxial compression tests and by consulting relevant literature [28,29,30,31,32,33]. The calibration targets were macroscopic mechanical properties of concrete, mainly the elastic modulus (*E*) and the axial compressive strength (*f*_cu_). The parameter system comprises two parts:Particle parameters: particle radii of cement mortar and CA (*R*_1_, *R*_2_); material density (*ρ*); particle elastic modulus (*E*_c_); and inter-particle friction coefficient (μ).

Parallel-bond parameters: to capture meso-scale behavior, a parallel-bond model was introduced at particle contacts. Key parameters include contact stiffness (normal *k_n_*, shear *k_s_*), bond strength (tensile bond strength *σ_b_* and cohesion *σ_c_*), and frictional properties (friction angle φ).

The material density (ρ) was assigned directly according to the measured density of the concrete. All key parameters are summarized in Table 5. Together, this parameter system enables the PB model to reproduce, under quasi-static loading, the meso-scale stress transfer, crack evolution, and stress distribution of cement-based materials with high fidelity.

(4) Unified constitutive model and calibration

All contacts adopt a unified linear parallel-bond (PB) constitutive model to ensure consistency of the modeling framework. During calibration, to reproduce the experimentally observed phenomenon that the ITZ contribution weakens at high CGS replacement, we slightly down-scaled the micro-parameters for CGS-involving interface contacts (including CGS–CA and CGS–NS)—specifically the tensile bond strength *σ_b_* and cohesion *σ_c_*—so that they are somewhat lower than those of NS-related contacts. This strategy preserves agreement with the overall stress–strain curves while more accurately capturing the macroscopic mechanical features at high CGS contents.

(5) Definition, classification, and counting of microcracks

Microcracks were quantified based on the state of PFC parallel bonds. A microcrack event was defined when any parallel-bonded contact transitioned from bonded to unbonded. The cumulative number of microcracks was normalized by the 2D specimen area (100 mm × 100 mm) to obtain the areal microcrack density, which served as the basis for comparisons among mixes. According to failure location, microcracks were further classified as Adhesive (interfacial debonding at particle–CA or particle–particle interfaces) and Cohesive (intra-particle fractures whose path lies entirely within the solid particle). The counts of both categories were accumulated separately and concurrently throughout loading.

#### 2.2.3. Parameter Calibration of the Model

**Figure 8:**
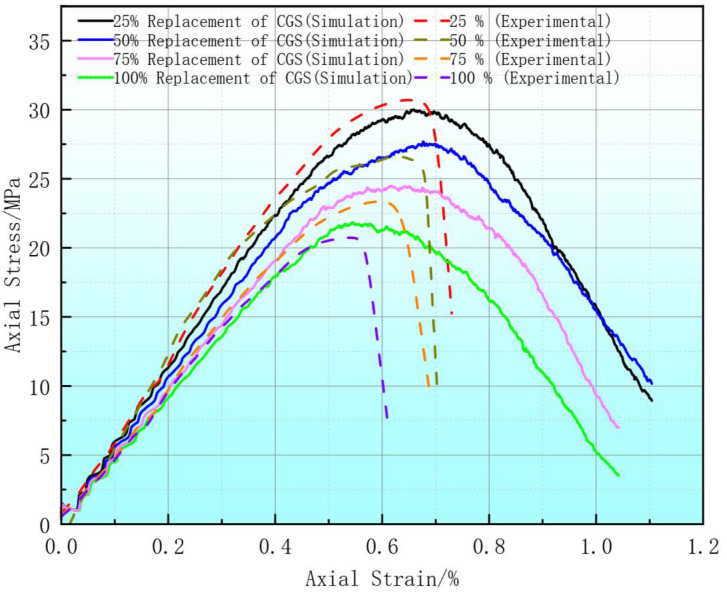
Simulated and experimental stress–strain.

The comparison of stress–strain curves at different CGS replacement rates (Figure 8) indicates that the PFC2D simulation results are in close agreement with the uniaxial compression test data. The errors in peak stress and strain are mostly within 5%, and the overall trend shows no significant deviation. The simulation curves closely match the experimental results in terms of the ascending slope during the elastic phase and the pre-peak stage. Although the post-peak descent is slightly more gradual in the simulation, it still maintains good consistency with the observed data. This demonstrates that the constructed model possesses high simulation accuracy and strong engineering applicability, effectively reflecting the mechanical behavior of coal CGS concrete and providing a reliable basis for subsequent studies on microscopic mechanisms.

#### 2.2.4. PFC2D-Based Numerical Simulation Scheme

Based on the validated model, a series of PFC2D simulations were conducted to further investigate the effects of CGS content on the mechanical behavior and failure mechanisms of concrete specimens. Concrete models with different CGS replacement ratios (25%, 50%, 75%, and 100%) were constructed. A quasi-static uniaxial compression loading mode was adopted, with the loading rate set to 0.01 mm/s.

The simulations aimed to compare the differences in force chain evolution, crack distribution, and energy dissipation among specimens with varying CGS contents. The specific simulation parameters for each group are summarized in Table 6.

## 3. Results

### 3.1. Analysis of Peak Stress and Strain

The strength performance of concrete is a critical indicator for evaluating its structural stability and load-bearing capacity, and it is typically quantified through the peak stress and its corresponding strain. Peak stress reflects the maximum load-carrying capacity of the material under external loading, whereas peak strain represents its deformation capacity prior to reaching the ultimate state [17,41]. The combination of these two parameters not only reveals the strength reserve and ductility characteristics of concrete but also provides a fundamental basis for investigating its performance degradation mechanisms and mechanical response behavior.

**Figure 9:**
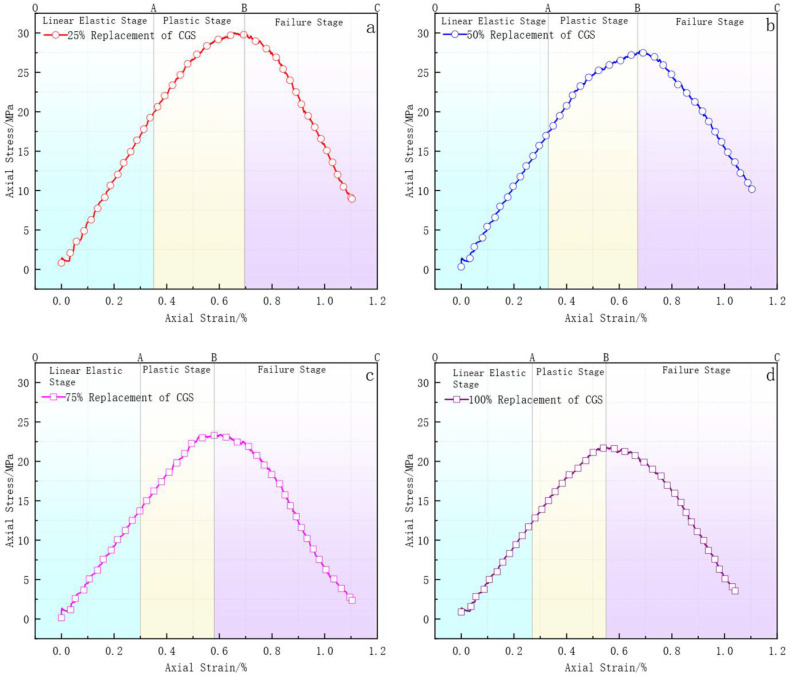
Stress–strain curve characteristics of specimens under a loading rate of 0.01 mm·s^−1^: (**a**) 25% coal gangue sand replacement; (**b**) 50% coal gangue sand replacement; (**c**) 75% coal gangue sand replacement; (**d**) 100% coal gangue sand replacement.

Figure 9a–d illustrates the stress–strain curves of concrete specimens with different CGS replacement ratios (25%, 50%, 75%, and 100%) under a loading rate of 0.01 mm/s. Overall, all specimens exhibited a typical three-stage mechanical response: (1) the linear elastic stage (OA segment), where the curve initially rises almost linearly in accordance with Hooke’s law; (2) the plastic stage (AB segment), during which the curve continues to increase but with a gradually reduced slope and slight fluctuations until reaching the peak; and (3) the degradation stage (BC segment), characterized by a sharp post-peak drop, indicating rapid deterioration of the structure and a significant reduction in load-bearing capacity.

For instance, the 25% replacement specimen reached a peak stress of nearly 30 MPa with a corresponding strain of approximately 0.9%, showing the highest load-carrying capacity and deformation ability among the four groups. The post-peak descending branch was relatively gradual, reflecting certain ductile features and a lower degree of performance degradation. With increasing replacement ratios, the 50% group showed slightly reduced peak stress but comparable peak strain, whereas the 75% and 100% groups exhibited earlier peak occurrence. In particular, the 100% replacement specimen showed the lowest peak stress and the steepest descending curve, indicating a progressive loss of ductility and a pronounced intensification of performance degradation.

This trend primarily originates from the inferior mechanical properties of CGS. Its elastic modulus (approximately 15–30 GPa) [11] is significantly lower than that of NS (35–50 GPa) [10], and the compressive strength of some particles is less than 10 MPa [9]. As the replacement ratio increases, the proportion of weak particles rises, leading to a weakened aggregate skeleton, increased porosity, and degraded interfacial bonding performance, thereby accelerating the overall performance degradation of concrete.

In summary, the CGS replacement ratio exerts a significant influence on the strength and deformation behavior of concrete. At low replacement levels (25% and 50%), concrete maintains relatively high load-bearing capacity and ductility, with higher peak values and smoother post-peak declines, reflecting a lower degree of degradation. By contrast, high replacement levels (75% and 100%) result in earlier peak occurrence, abrupt stress drops, and aggravated brittle degradation. The underlying reason lies in the insufficient mechanical performance of CGS, which causes a loose skeleton structure and weakened interfacial bonding, thereby undermining continuous load-transfer paths and accelerating the degradation process.

### 3.2. Force Chain Analysis

Force chains, formed through particle–particle contact interactions, constitute the primary stress-bearing pathways in concrete. They play a critical role in transmitting and redistributing internal stresses and serve as a vital link between meso-scale structures and macroscopic mechanical performance [18,19]. During loading, the formation, breakage, and reorganization of force chains directly determine stress concentration zones and the pathways of degradation evolution. A continuous and uniformly distributed force-chain network helps maintain structural stability and ductility, whereas the occurrence of breakages or voids often indicates that the material has entered a stage of performance degradation [15,30]. Therefore, the evolution of force chains provides an important basis for elucidating the micro-mechanisms of performance degradation in CGS concrete.

**Figure 10:**
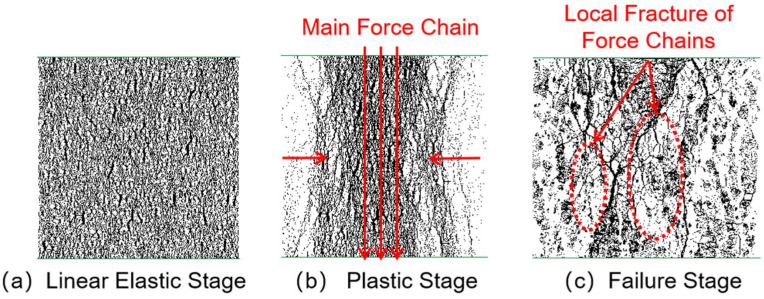
Force chain distribution of specimen with 25% coal gangue sand replacement.

**Figure 11:**
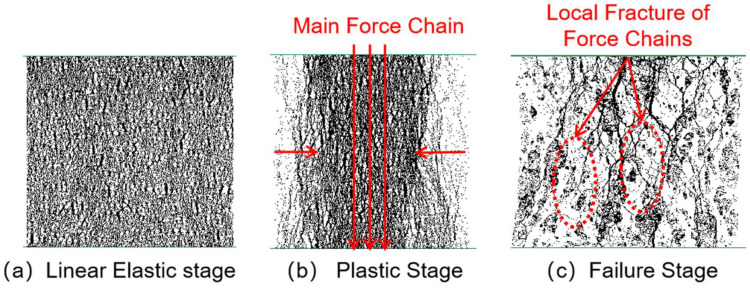
Force chain distribution of specimen with 50% coal gangue sand replacement.

**Figure 12:**
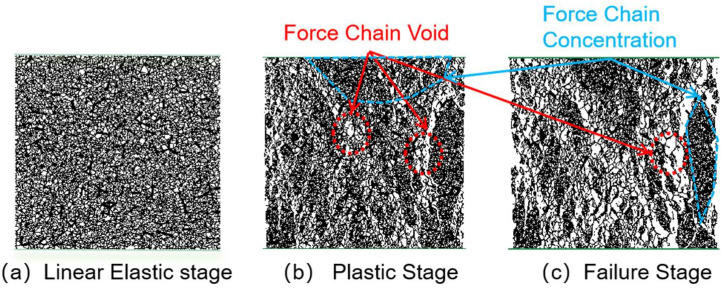
Force chain distribution of specimen with 75% coal gangue sand replacement.

**Figure 13:**
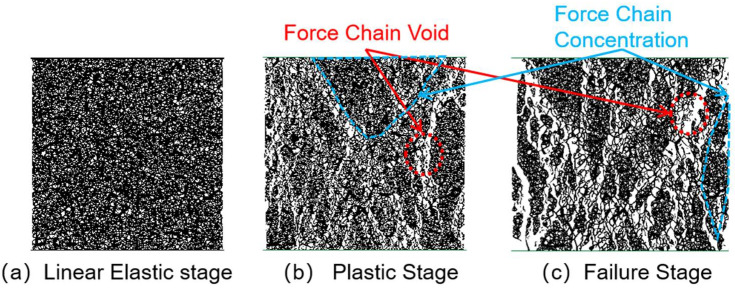
Force chain distribution of specimen with 100% coal gangue sand replacement.

Figure 10, Figure 11, Figure 12 and Figure 13 illustrate the force-chain evolution characteristics of specimens with different CGS replacement ratios during loading. Taking the 25% replacement group as an example, in the linear elastic stage, force chains were evenly distributed and exhibited pronounced isotropy (Figure 10a). Entering the plastic stage, local breakages began to occur on both sides of the specimen, while the main force chains gradually clustered in the vertical direction, but overall continuity was still preserved (The orientation of force chains is indicated by the arrows in Figure 10b). In the degradation stage, local voids appeared within the main force chains, which experienced partial breakages, although secondary stress-transfer paths remained, providing limited ductility and residual load-bearing capacity (Figure 10c).

In comparison, the 50% replacement group exhibited a similar overall trend to the 25% group, but more pronounced breakages and voids were observed in both the plastic and degradation stages (The orientation of force chains is indicated by the arrows in Figure 11b,c), reflecting a further reduction in ductility. The 75% and 100% replacement groups showed markedly different behavior: local clustering of force chains occurred even in the early loading stage; in the plastic stage, the originally continuous force-chain system progressively degraded into short-chain structures, shifting from overall connectivity to localized distribution, with voids concentrated along the specimen sides and central region. By the degradation stage, voids in the central region became highly dense, and breakage zones rapidly penetrated from both sides toward the center, while most other regions nearly lost their stress-transfer capacity, indicating that the structure had entered a state of severe performance degradation (Figure 12 and Figure 13b,c).

In summary, the CGS replacement ratio exerts a significant influence on the integrity and stability of internal force-chain structures in concrete. At low replacement levels (25–50%), force chains were uniformly distributed and well-connected, enabling efficient stress transfer and imparting greater ductility and residual strength. At higher replacement levels (75–100%), force chains became increasingly localized, connectivity was weakened, and voids and breakages intensified, ultimately inducing typical brittle degradation. The observed evolution of force chains corresponds well with the stress–strain responses, revealing the meso-scale mechanism underlying the transition of CGS concrete from ductile to brittle degradation, and providing important theoretical support for determining appropriate replacement ratios and optimizing concrete mix designs.

### 3.3. Crack Analysis

Cracks are a direct manifestation of internal performance degradation in materials. Their evolution reflects stress redistribution and localized degradation characteristics in concrete under loading, while also revealing the fracture pathways of force-chain networks and the mechanisms of structural deterioration [18,27]. During loading, progressive instability of force chains leads to stress concentration, which in turn promotes crack initiation and propagation, ultimately penetrating the main force chains and resulting in an overall reduction in structural performance. Thus, crack evolution not only reflects differences in ductility and brittleness but also maps the stability of force-chain structures [30]. This section analyzes the micro-mechanisms of performance degradation in CGS concrete under different replacement ratios from the perspectives of crack propagation patterns and spatial distribution.

#### 3.3.1. Crack Propagation Analysis

**Figure 14:**
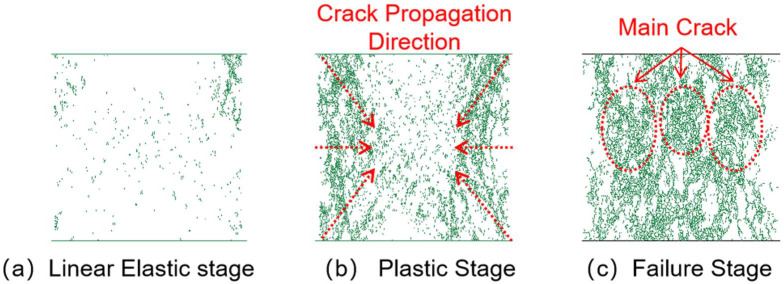
Crack distribution of specimen with 25% coal gangue sand replacement.

**Figure 15:**
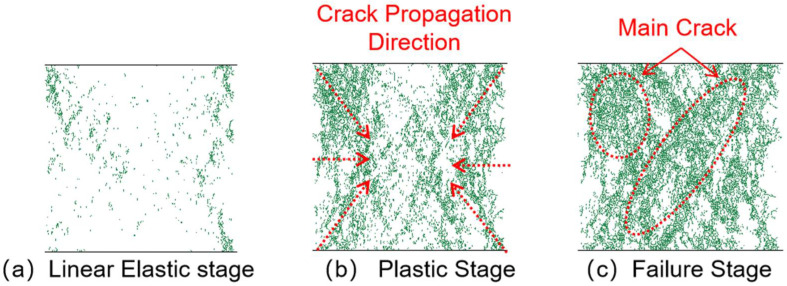
Crack distribution of specimen with 50% coal gangue sand replacement.

**Figure 16:**
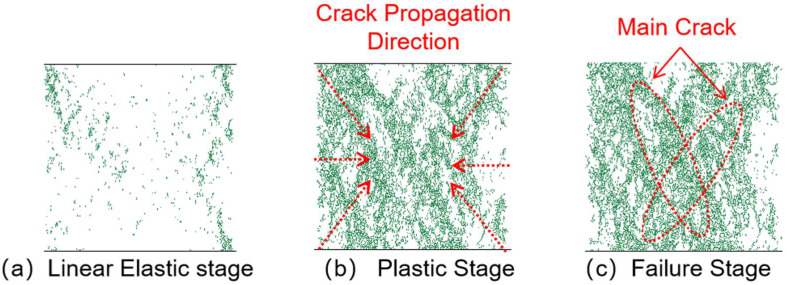
Crack distribution of specimen with 75% coal gangue sand replacement.

**Figure 17:**
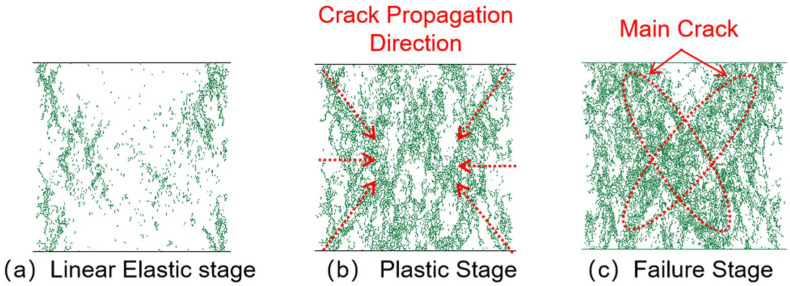
Crack distribution of specimen with 100% coal gangue sand replacement.

Figure 14, Figure 15, Figure 16 and Figure 17 show the crack distribution characteristics of concrete specimens with different CGS replacement ratios (25–100%) during the loading process. For the 25% replacement group, in the linear elastic stage (Figure 14a), the specimen remained largely intact with only a few initial cracks appearing at the edges, indicating that the stress was insufficient to trigger significant local degradation. Entering the plastic stage (Figure 14b), primary cracks gradually developed along both sides, accompanied by a few secondary cracks extending toward the center. The relatively dispersed crack distribution demonstrated the specimen’s capacity to release and buffer stress. In the degradation stage (Figure 14c), a primary crack propagated along the loading axis but did not fully penetrate, while secondary cracks were still present. The damage zone was not highly concentrated, and the specimen maintained certain ductility and residual load-bearing capacity.

By comparison, the 50%, 75%, and 100% replacement groups exhibited similar crack initiation and propagation processes but with significant differences in density and connectivity. The 50% replacement group showed a tendency toward crack concentration in the plastic stage, and by the degradation stage, the number of cracks increased significantly, with localized diagonal cracks penetrating through the specimen (Figure 15b,c). The 75% and 100% replacement groups exhibited more typical brittle degradation behavior: multiple dense cracks formed as early as the plastic stage, and during the degradation stage, primary cracks rapidly penetrated the specimen. Crack spacing was substantially reduced, and crack bands became highly concentrated (Figure 16 and Figure 17b,c).

**Figure 18:**
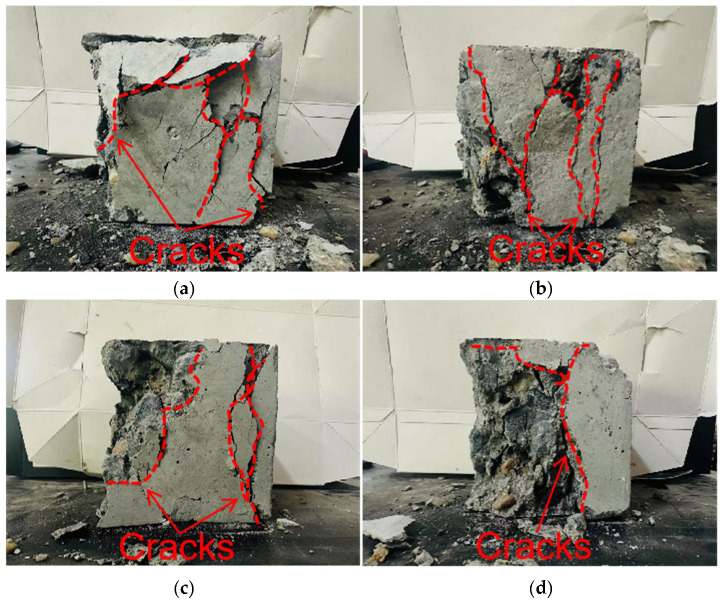
Macroscopic failure patterns of specimens under different coal gangue sand replacement rates: (**a**) 25% replacement rate of CGS, (**b**) 50% replacement rate of CGS, (**c**) 75% replacement rate of CGS, (**d**) 100% replacement rate of CGS.

To further compare degradation modes under different replacement ratios, Figure 18 presents the final crack morphologies of specimens subjected to uniaxial compression. In the 25% replacement group (Figure 18a), cracks were discretely distributed without full penetration, and structural integrity was largely preserved, indicating strong crack resistance consistent with the uniform and continuous force-chain network. In the 50% group (Figure 18b), the number of cracks increased, and localized diagonal cracks appeared, reflecting reduced ductility due to force-chain breakages and void formation during the plastic and degradation stages. In the 75% and 100% groups (Figure 18c,d), specimens exhibited a typical splitting degradation mode, with cracks rapidly penetrating along fixed paths, resulting in highly concentrated crack bands and severely compromised structural integrity. These observations are consistent with the early clustering of force chains, dense void formation, and discontinuous stress-transfer pathways.

In summary, the CGS replacement ratio exerts a significant influence on crack evolution and degradation modes of concrete. At low replacement levels (25% and 50%), cracks initiated slowly, were relatively dispersed, and the primary cracks were difficult to fully penetrate, indicating better ductility and toughness. This was mainly attributed to stable force-chain networks and uniform stress distribution. At high replacement levels (75% and 100%), cracks developed intensively from the plastic stage and rapidly penetrated during degradation, exhibiting a typical brittle degradation mode. These results demonstrate that as the replacement ratio increases, force-chain networks progressively destabilize, stress concentration intensifies, and cracks propagate rapidly, ultimately accelerating macroscopic performance degradation.

#### 3.3.2. Analysis of Particle-Contact Cracks

To clearly distinguish crack types and origins, particle-contact cracks are classified into Cohesive (intra-particle) cracks and Adhesive (interfacial) cracks. The former occur within a homogeneous particle (e.g., within a CGS or natural-sand particle), whereas the latter are located at heterogeneous particle interfaces (e.g., the contact between CGS and NS) and are markedly enriched in the interfacial transition zone (ITZ) between aggregate and mortar [19]. Affected by fluctuations in water–binder ratio, phase discontinuities, and elevated porosity, the ITZ generally exhibits lower shear and tensile resistance than the aggregate or cementitious matrix, thereby becoming a weak zone prone to stress concentration and preferential microcrack initiation.

**Figure 19:**
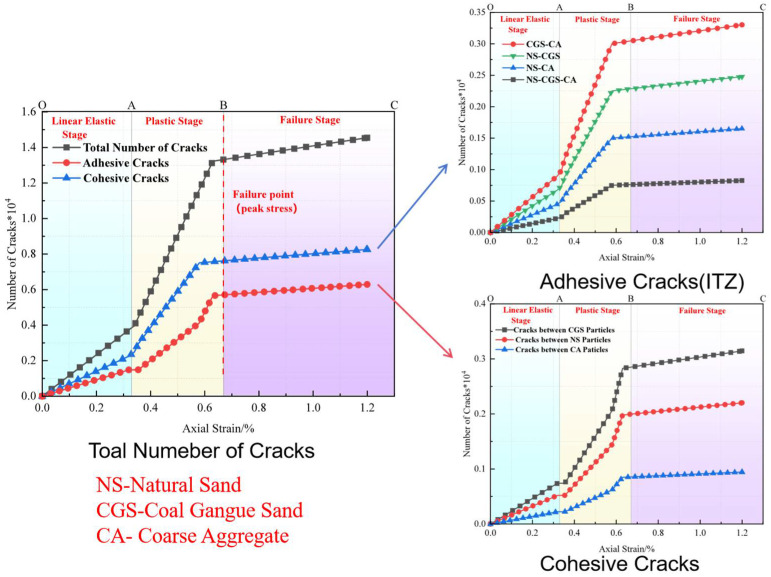
Classification and number of cracks in specimens utilizing 25% CGS.

**Figure 20:**
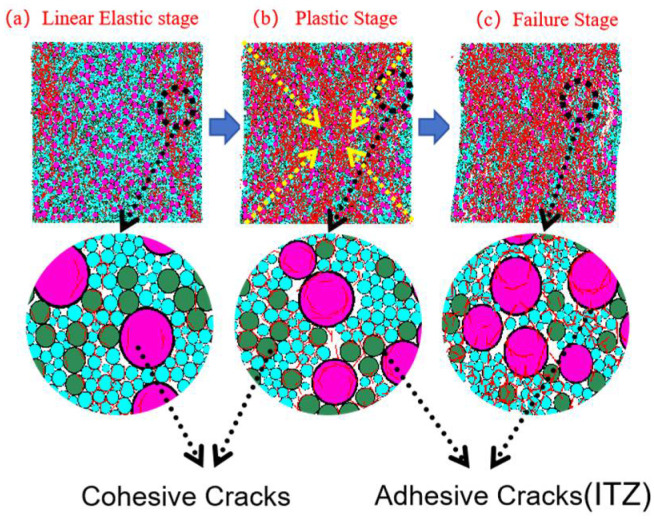
Distribution of internal cracks within a 25% CGS blend specimen.

**Figure 21:**
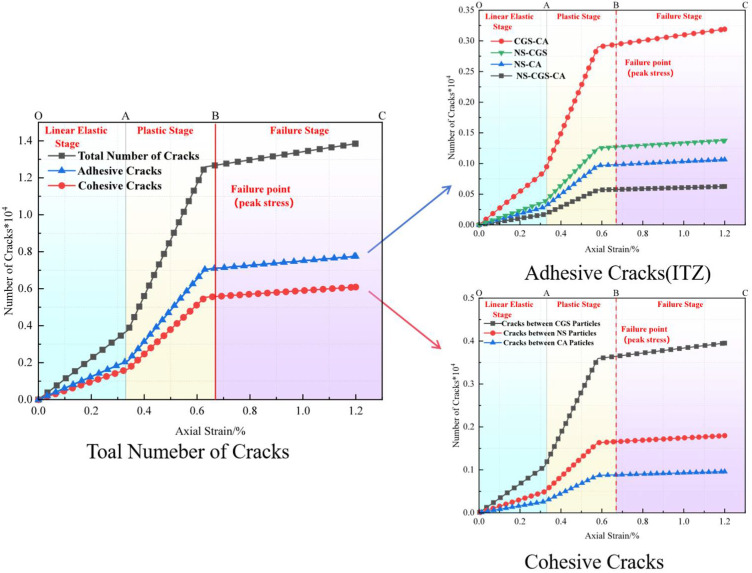
Classification and number of cracks in specimens utilizing 50% CGS.

**Figure 22:**
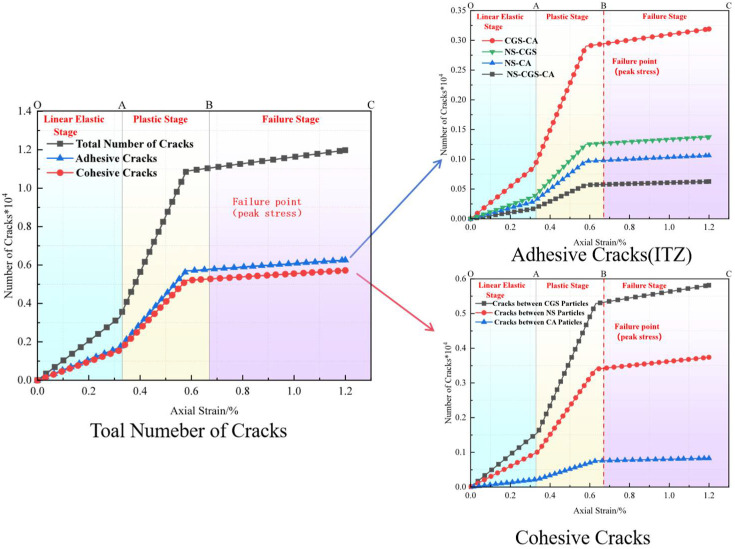
Classification and number of cracks in specimens utilizing 75% CGS.

**Figure 23:**
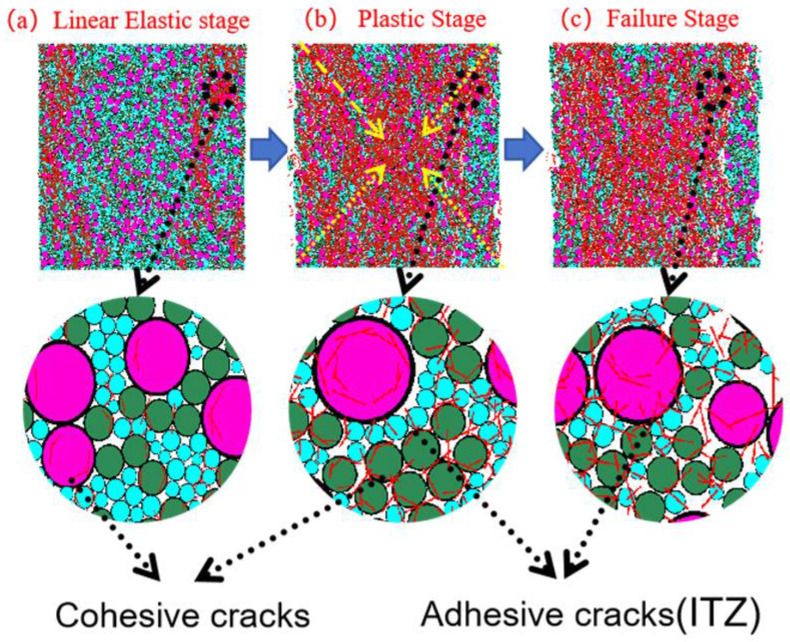
Distribution of internal cracks within a 50% CGS blend specimen.

**Figure 24:**
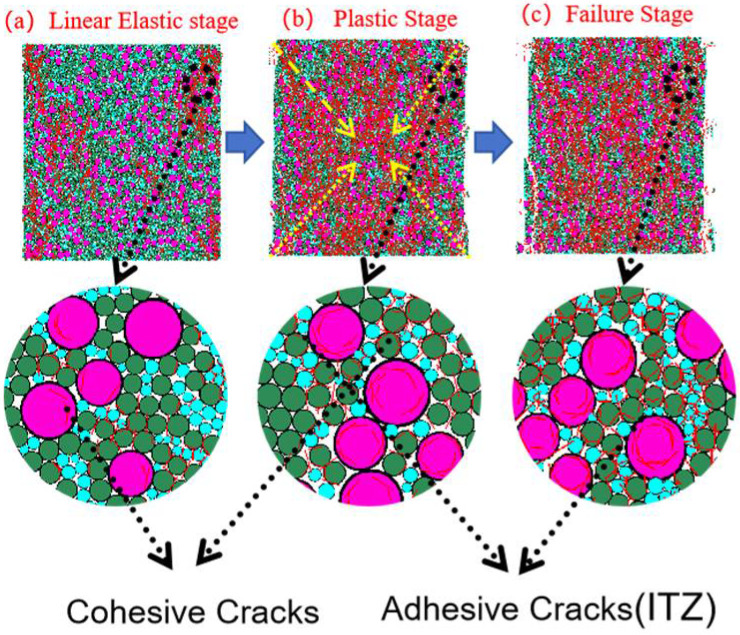
Distribution of internal cracks within a 75% CGS blend specimen.

**Figure 25:**
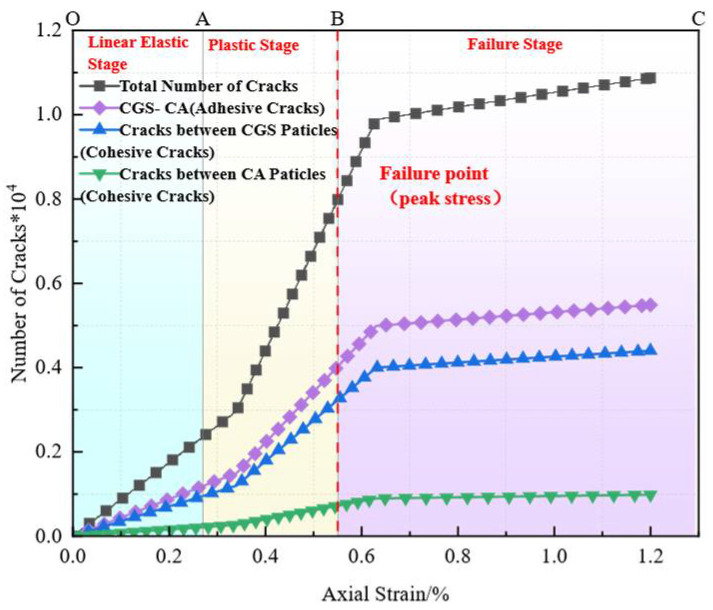
Classification and number of cracks in specimens utilizing 100% CGS.

**Figure 26:**
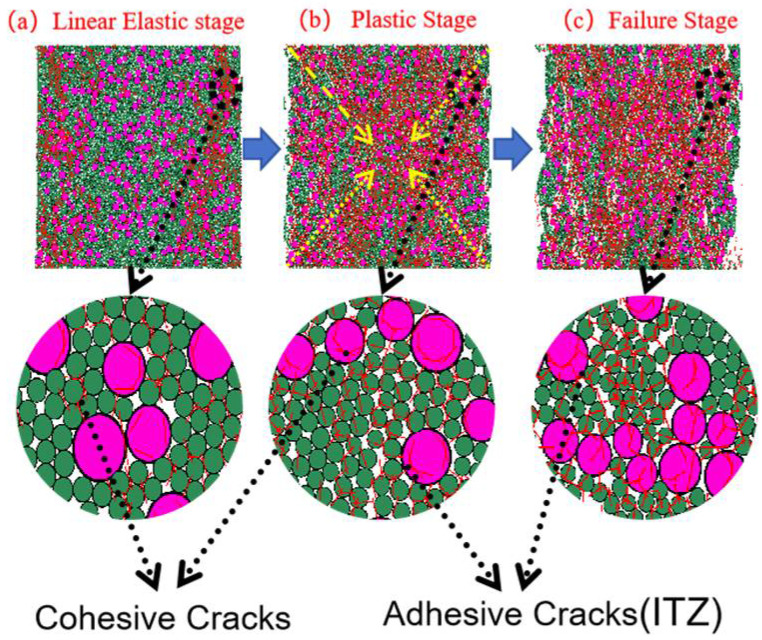
Distribution of internal cracks within a 100% CGS blend specimen.

To characterize crack evolution during loading, we counted, at a given axial strain ε, the cumulative number of failures of same-phase and different-phase particle contacts, and obtained the normalized cumulative crack count *N*(*ε*) (normalized by the 2D specimen area of 100 mm × 100 mm). We then plotted the evolution of total cracks, Adhesive (ITZ) cracks, and Cohesive cracks versus strain. In parallel, we provided spatial crack maps and representative microstructural snapshots at three characteristic stages—elastic, plastic, and failure—to enable cross-validation from both “count” and “spatial” perspectives (Figure 19, Figure 20, Figure 21, Figure 22, Figure 23, Figure 24, Figure 25 and Figure 26).

Figure 19, Figure 20, Figure 21 and Figure 22 present the crack counts and spatial distributions for four CGS replacement ratios (25%, 50%, 75%, 100%). Taking the 25% group as an example (Figure 19), crack evolution can be divided into three stages: Elastic stage (OA): both Adhesive and Cohesive crack counts are low and increase slowly; Adhesive cracks account for ~35% of total cracks, mainly distributed along two-phase ITZ (NS–CGS) (Figure 20a), with a small fraction at three-phase ITZ (NS–CGS–CA). The specimen remains structurally continuous, indicating a relatively isotropic, well-distributed force-chain network that disperses stress and suppresses early cracking. Plastic stage (AB): the total crack count rises rapidly with a pronounced inflection; the fraction of Adhesive cracks exceeds 55%, still dominated by two- and three-phase ITZ. Cohesive cracks occur primarily within natural-sand particles; cracks expand from the edges toward the center but are not yet coalesced (Figure 20b). Failure stage (BC): after reaching the peak stress (failure point), both Adhesive and Cohesive cracks approach saturation, become widely distributed, and are accompanied by void formation; load-bearing capacity decreases significantly (Figure 20c).

As the CGS replacement increases (50%, 75%, 100%), the main meso-structural changes relative to 25% are as follows. 50% group (Figure 21 and Figure 23): Changes in particle gradation and phase composition led to a slight decrease in the total crack count. The reduction in natural sand content and the increase in CGS content resulted in a decrease in the number of three-phase ITZs, causing adhesive cracks to concentrate more at the NS–CGS two-phase interfaces. The deterioration of the ITZ contributed to a decreased proportion of adhesive cracks, which initiated earlier and more readily; meanwhile, the proportion of cohesive cracks increased correspondingly. 75% and 100% groups (Figure 22, Figure 24, Figure 25 and Figure 26): The number of three-phase ITZs further decreased. Adhesive cracks were mainly distributed along the CGS–CA two-phase interfaces, and their proportion in the total cracks continued to decline; in contrast, the proportion of cohesive cracks within CGS particles increased significantly from the late plastic stage into the post-peak stage. At high replacement levels, increased porosity and degraded interfacial quality jointly undermined structural integrity, promoting the formation and development of a crack network dominated by two-phase adhesive cracks and cohesive cracks within CGS particles. This process explains the observed localization of force chains, increased intermittency, shortened and less repeatable load paths, and, macroscopically, reduced ductility and enhanced brittleness.

### 3.4. Scanning Electron Microscopy (SEM) Analysis

**Figure 27:**
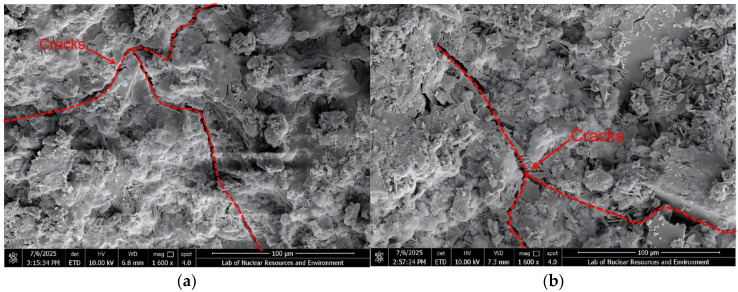

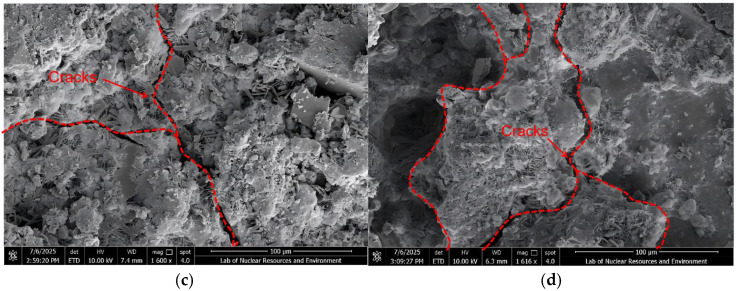
Microscopic cracks in the specimen under 1600× SEM magnification: (**a**) 25% replacement rate of CGS, (**b**) 50% replacement rate of CGS, (**c**) 75% replacement rate of CGS, (**d**) 100% replacement rate of CGS.

**Figure 28:**
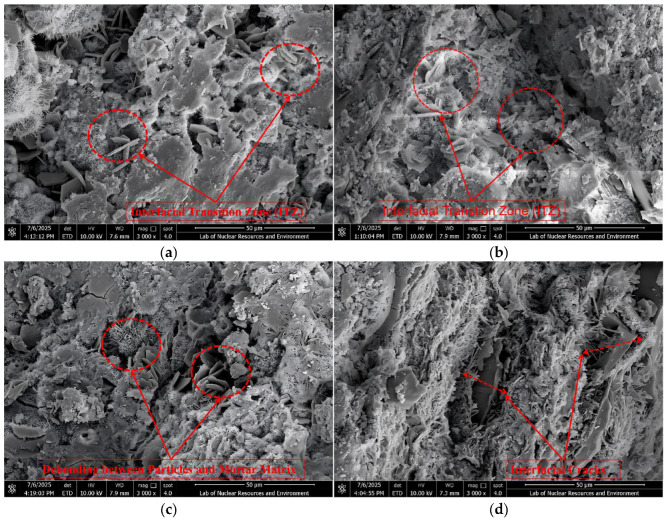
Microscopic cracks in the specimen under 3000× SEM magnification: (**a**) 25% replacement rate of CGS, (**b**) 50% replacement rate of CGS, (**c**) 75% replacement rate of CGS, (**d**) 100% replacement rate of CGS.

To systematically investigate interfacial characteristics at different CGS replacement levels, representative specimens were examined by SEM (Figure 27 and Figure 28). The ITZ was interpreted from two perspectives: interfacial phase architecture and pore/crack morphology.

#### 3.4.1. ITZ Classification Criteria and SEM Observations

Following prior studies on ITZ characterization [42,43,44], the criteria adopted herein are: Three-phase ITZ (NS-CGS-CA): a continuous transitional layer is present at the interface and all of the following are satisfied: (1) hydration products are dense and uniformly distributed; (2) porosity is low and no through-cracks are present; (3) Aggregate-mortar bonding is good; Two-phase ITZ (direct two-phase contact, e.g., CGS-CA): the interface is dominated by direct contact between two phases and at least three of the following four features are present: (1) evident pores or interfacial gaps; (2) observable interfacial debonding; (3) cracks with a tendency to penetrate/coalesce; (4) discontinuous or porous hydration products.

These criteria were applied consistently across all replacement levels in SEM image interpretation. At 1600× magnification (Figure 27), low-replacement specimens exhibit narrow cracks with an intact surrounding matrix (Figure 27a,b), whereas high-replacement specimens show significantly wider cracks, often accompanied by voids or interfacial gaps during propagation (Figure 27c,d). At 3000× magnification, further inspection of crack-growth zones and ITZ features reveals: for ≤50% replacement, CGS fills and bridges the voids between NS and CA, forming a three-phase ITZ. The interface appears dense with low porosity, and most microcracks remain closed (Figure 28a,b), which suppresses early crack initiation and helps maintain toughness. When the replacement increases to ≥75%, many regions transform into two-phase ITZ (CGS-CA) with pronounced interfacial debonding/gaps and higher porosity (Figure 28c,d); load-transfer paths shorten and become localized, cracks preferentially propagate along weak interfaces, and macroscopic degradation accelerates.

#### 3.4.2. Correlation Between SEM Observations and DEM Simulations

The SEM observations and DEM numerical simulations demonstrate strong consistency at the meso-scale, collectively revealing the micro-mechanisms behind the variations in concrete performance under different CGS replacement ratios. Based on the morphology and structural characteristics of the interfacial transition zone observed via SEM, this study correspondingly implemented differentiated contact parameters in the DEM modeling: for the 25% CGS group, which exhibited a “dense three-phase ITZ” in SEM images, higher bond stiffness and fracture strength were assigned in the model to reflect its superior interfacial bonding properties; whereas for the 100% CGS group showing a “porous two-phase ITZ”, the interfacial contact parameters were appropriately reduced to simulate its weaker bonding behavior and higher tendency for micro-crack initiation.

Specifically, under low replacement ratios (≤50%), the DEM simulations showed that micro-cracks were dispersed and accumulated slowly, while the force chain networks remained relatively continuous. These findings are consistent with the dense three-phase ITZ structure observed in SEM images, indicating that this ITZ configuration effectively provides bridging and filling effects, thereby maintaining efficient stress transfer. In contrast, under high replacement ratios (≥75%), the DEM results indicated accelerated crack accumulation and noticeable localization. SEM further confirmed that a two-phase ITZ (direct CGS–CA contact) dominates under these conditions, characterized by higher porosity and weakened interfacial bonding. This leads to increased crack susceptibility and deterioration of force transmission paths, which macroscopically manifests as reduced load-bearing capacity and enhanced brittleness.

### 3.5. Energy Evolution Analysis

During the performance degradation of particulate materials such as concrete, the macroscopic mechanical response essentially originates from internal force transfer, structural reorganization, and energy transformation within the particle system [32]. Unlike continuum media, in discrete particle systems subjected to external loading, energy is gradually accumulated locally as strain energy and continuously dissipated through inter-particle frictional sliding and viscous damping. When local force chains break and cause stress redistribution, cracks tend to initiate in regions of stress concentration and propagate along the paths of the main force chains, ultimately leading to system performance degradation [20,32,33]. Therefore, energy accumulation and release not only drive the degradation evolution but are also closely linked to crack propagation behavior and force-chain stability, serving as key physical parameters for elucidating the micro-mechanisms of concrete degradation.

#### 3.5.1. Energy Evolution Patterns Under Different Replacement Ratios

To gain deeper insight into the energy response mechanisms of CGS concrete during the loading process, this study extracted and analyzed the energy components of the particle system using the PFC2D platform. Real-time acquisition of each energy component throughout the uniaxial compression process was achieved through the FISH programming language. The monitored energy terms include: Total Input Energy, Bond Energy (*E_bound_*), Strain Energy (*E_strain_*), Frictional Energy (*E_fric_*), Damping Energy (*E_damp_*), and Kinetic Energy (*E_kin_*). Specifically, Total Energy represents the cumulative external energy applied to the specimen during loading. *E_bound_* denotes the energy dissipated due to the breakage of bonded contacts between particles, highlighting the dominant role of interfacial damage in the structural failure process. *E_strain_* reflects the structure’s capacity to store elastic energy. *E_fric_* and *E_damp_* represent energy dissipation caused by inter-particle frictional sliding and viscous damping, respectively. *E_kin_*, under quasi-static loading conditions, is negligible and thus not considered in subsequent analyses.

**Figure 29:**
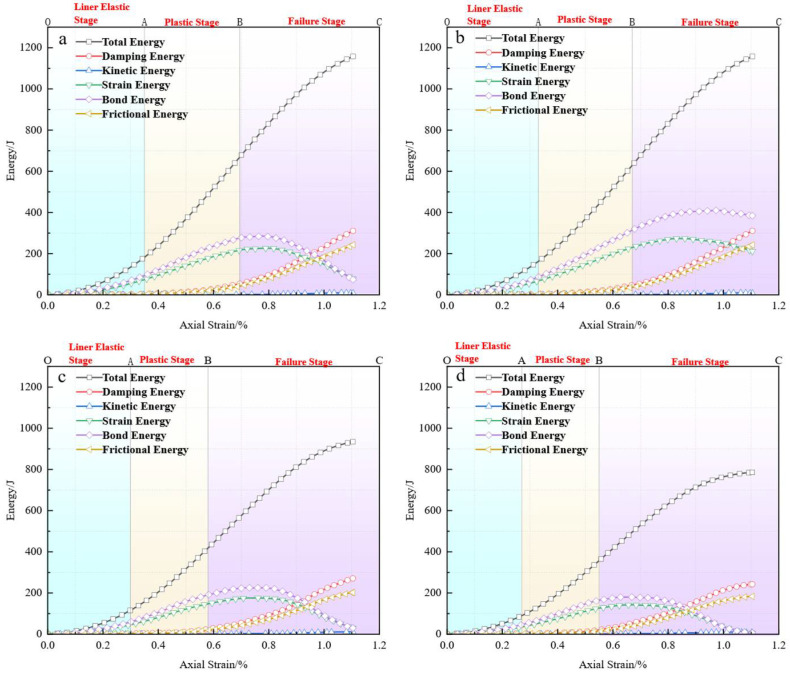
Energy evolution curves under different coal gangue sand replacement rate: (**a**) 25% coal gangue sand replacement; (**b**) 50% coal gangue sand replacement; (**c**) 75% coal gangue sand replacement; (**d**) 100% coal gangue sand replacement.

Figure 29 presents the energy evolution curves for specimens with different CGS replacement ratios. Taking the 25% replacement group as an example (Figure 29a), the energy evolution can be divided into three stages: in the linear elastic stage (OA), *E_bound_* and *E_strain_* increase approximately linearly, with energy stored elastically at particle contacts, and the main force chains carry the majority of stress transfer without observed crack formation; in the plastic stage (AB), the growth rate of *E_strain_* slows, while *E_fric_* and *E_damp_* continue to rise, indicating intensified particle sliding and energy dissipation, accompanied by crack initiation and local propagation, partial force-chain breakage and reorganization, and localized stress concentrations; in the failure stage (BC), Estrain peaks and then declines, while *E_fric_* and *E_damp_* rise sharply, marking a transition to a dissipation-dominated stage, with cracks clustering and propagating along the main force-chain paths, the force-chain skeleton severely degraded but retaining some residual load-bearing capacity, exhibiting limited ductility.

A comparison across different replacement ratios (Figure 29b–d) reveals that although the general pattern of energy evolution remains consistent, significant quantitative differences exist. With increasing CGS replacement, the peak value of *E_strain_* progressively decreases, while the proportions of *E_fric_* and *E_damp_* in the total energy increase markedly, indicating a weakened energy storage capacity of the system and enhanced contributions from frictional and damping dissipation. This characteristic aligns well with the previously identified failure mechanism of “concentrated crack initiation, rapid penetration, and force-chain instability.” Specifically, at low replacement levels (25%, 50%), cracks are discretely distributed, force chains remain well-connected, energy is stored primarily in elastic form, and the dissipation process is relatively gradual, resulting in favorable ductility and degradation resistance. At high replacement levels (75%, 100%), cracks appear intensively at an early loading stage and rapidly penetrate, force chains cannot be effectively reconstructed, and energy consumption is dominated by frictional and damping dissipation, leading to a typical brittle failure mode.

#### 3.5.2. Relationship Between Energy Evolution and Performance Degradation Mechanisms

The CGS replacement ratio influences the failure mode and macroscopic performance of concrete by altering the balance between energy storage and dissipation within the system. At low replacement levels, *E_strain_* dominates, the system exhibits strong energy storage capacity, *E_fric_* and *E_damp_* increase moderately, crack propagation is slow, force-chain structures remain stable, and the material demonstrates good ductility. At high replacement levels, the peak *E_strain_* decreases, while the proportions of *E_fric_* and *E_damp_* rise significantly, making the system more reliant on interfacial sliding for energy dissipation, promoting concentrated crack penetration and force-chain instability, and ultimately resulting in brittle failure. The evolution of energy distribution characteristics is consistent with the crack propagation process and force-chain degradation behavior, collectively revealing the energy-driven mechanism behind the performance degradation of CGS concrete.

## 4. Discussion

This study systematically investigated the degradation mechanisms of coal CGS concrete under varying replacement ratios through an integrated approach combining uniaxial compression tests, PFC2D discrete element simulations, and scanning electron microscopy (SEM) observations. The results demonstrate that the CGS replacement ratio serves as a critical factor governing the transition from ductile to brittle failure modes. Detailed conclusions are systematically summarized in the Conclusions section and will not be reiterated here. During the investigation, several limitations were identified, which provide valuable directions for future research.

### 4.1. Limitations of the Study

Although this multi-scale approach successfully revealed the performance degradation mechanisms of CGS concrete, several limitations warrant further consideration. First, while the adopted PFC2D model effectively captured the mechanical behavior under plane stress conditions, it cannot fully represent the complex mechanical responses in actual three-dimensional stress states, particularly the evolution patterns of force-chain networks under multi-axial stress conditions. Second, although the trial-and-error method employed in meso-parameter calibration yielded parameter sets that generally align with macroscopic test results, this approach requires improvement in terms of parameter identifiability and optimality. Additionally, this study primarily focused on static mechanical properties, without adequately accounting for the influences of complex factors such as dynamic loading and environmental temperature variations encountered in practical engineering applications. Notably, the simulation of the interfacial transition zone (ITZ) in PFC2D lacks clarity in representing the arrangement and evolution of the ITZ region, and there is an absence of quantitative indicators to accurately characterize its meso-scale features.

### 4.2. Future Research Directions

Building upon these limitations, future research will focus on the following key directions: In the area of meso-parameter optimization, we will prioritize the development of an automated DEM parameter calibration method based on multi-objective genetic algorithms (MOGA) [45]. Drawing on the successful experience of Santos et al. in their study on colored self-compacting concrete, we aim to establish a multi-objective optimization framework capable of simultaneously matching stress–strain responses, crack propagation patterns, and energy evolution characteristics. Advancements in this direction will significantly enhance the systematicity and reliability of parameter identification, providing technical support for establishing accurate meso-mechanical models.

Regarding the influence of complex environmental factors, subsequent work will emphasize investigating material degradation mechanisms under the coupled effects of wet-dry cycles [46], freeze–thaw damage [47], and chemical attacks [48]. Through systematically designed accelerated test protocols, we plan to thoroughly examine the synergistic effects of environmental factors and mechanical loading on ITZ structural evolution, force-chain network stability, and energy dissipation mechanisms. This research will contribute to the development of performance prediction models that better reflect actual engineering conditions.

Furthermore, we will dedicate efforts to developing quantitative characterization methods for the interfacial transition zone. By establishing quantitative relationships between ITZ microstructural parameters and macroscopic mechanical properties, we seek to enhance the predictive capability for material performance degradation. The in-depth exploration of these research directions will provide more reliable theoretical foundations and technical support for the engineering application of CGS concrete.

## 5. Conclusions

Based on the discrete element method (PFC2D), a two-dimensional meso-scale model of coal gangue manufactured sand (CGS) concrete was developed and coupled with uniaxial compression tests to systematically investigate how different CGS replacement levels (25%, 50%, 75%, 100%) affect the meso-mechanical response and degradation mechanisms, with emphasis on peak strength, force-chain evolution, crack propagation, and energy dissipation. The main conclusions are as follows:

(1) The numerical results agree closely with the uniaxial test data; for all mixes, the errors in peak stress and peak strain are <5%, confirming the reliability and applicability of the particle-flow model for capturing the degradation process of CGS concrete.

(2) The 25% replacement exhibits the best overall performance. During loading, the evolution of force chains, cracking, and energy components are coordinated and stable. The two- and three-phase ITZ formed at 25% provide stable load-transfer paths, effectively suppressing early crack growth and conferring strong resistance to degradation.

(3) As the CGS replacement increases (25–100%), the ITZ transforms from a dense three-phase ITZ (NS–CGS–CA) to a degraded two-phase ITZ (e.g., CGS–CA); the fraction of Adhesive cracks decreases while that of Cohesive cracks increases. The force-chain network degrades from a continuous structure into localized short-chain clusters, and the energy partition shifts from strain-energy storage to frictional/damping dissipation, corresponding to a macroscopic transition from ductile to brittle behavior.

(4) The concordance between SEM and DEM in crack spatial distribution, force-chain localization, and energy evolution substantiates the mechanistic chain “increasing replacement → ITZ degradation → force-chain instability/greater dissipation → performance loss.” For routine applications, a low-to-moderate replacement window (25–50%) is recommended to balance environmental benefits with mechanical performance.

## Figures and Tables

**Table 1:** Performance indicators of coal gangue mechanism sand.

Apparent Density (kg/m^3^)	Bulk Density (kg/m^3^)	Void Ratio (%)	Stone Powder Content (%)	Clay Lump Content (%)	Crushing Index (%)	Fineness Modulus
2700	1620	40	14.8	0.2	21	2.65

**Table 2:** Mix Proportions of Coal Gangue Sand Concrete.

Mix ID	Cement (kg/m^3^)	Water (kg/m^3^)	Coarse Aggregate (kg/m^3^)	Fine Aggregate (kg/m^3^)	
			Stone	Sand	CGS
J1	400	180	1120	510	170
J2	400	180	1120	340	340
J3	400	180	1120	170	510
J4	400	180	1120	0	680

**Table 3:** Mean peak stress and confidence intervals of coal gangue sand concrete at different curing ages.

Mix ID	Age	Mean ± SD/MPa	95% CI	n
J1	1 d	13.53 ± 0.35	[12.66–14.41]	3
J1	3 d	19.63 ± 0.53	[18.31–20.95]	3
J1	7 d	25.66 ± 0.65	[24.04–27.27]	3
J1	28 d	30.74 ± 0.65	[29.40–32.08]	3
J2	1 d	13.5± 0.2	[13.05–14.0]	3
J2	3 d	16.57± 0.41	[15.57–17.58]	3
J2	7 d	23.26 ± 0.98	[20.82–25.71]	3
J2	28 d	26.71 ± 0.72	[24.92–28.5]	3
J3	1 d	11.17± 0.1	[10.92–11.42]	3
J3	3 d	13.51 ± 0.32	[12.73–14.30]	3
J3	7 d	19.94± 0.24	[19.35–20.53]	3
J3	28 d	23.83± 0.68	[22.14–25.52]	3
J4	1 d	10.36± 0.37	[9.44–11.28]	3
J4	3 d	11.08± 0.56	[9.68–12.47]	3
J4	7 d	15.67 ± 0.49	[14.45–16.90]	3
J4	28 d	20.85± 0.7	[19.12–22.57]	3

**Table 4:** Mean peak strain and confidence intervals of coal gangue sand concrete at different curing ages.

Mix ID	Age	Mean ± SD/%	95% CI	n
J1	1 d	0.578 ± 0.008	[0.565, 0.597]	3
J1	3 d	0.591 ± 0.003	[0.584, 0.598]	3
J1	7 d	0.624 ± 0.006	[0.611, 0.639]	3
J1	28 d	0.673 ± 0.005	[0.661, 0.685]	3
J2	1 d	0.563 ± 0.008	[0.543, 0.584]	3
J2	3 d	0.576 ± 0.005	[0.563, 0.589]	3
J2	7 d	0.618 ± 0.01	[0.593, 0.643]	3
J2	28 d	0.655 ± 0.009	[0.634, 0.676]	3
J3	1 d	0.544 ± 0.005	[0.533, 0.555]	3
J3	3 d	0.546 ± 0.01	[0.522, 0.571]	3
J3	7 d	0.576 ± 0.007	[0.559, 0.594]	3
J3	28 d	0.616 ± 0.004	[0.605, 0.627]	3
J4	1 d	0.524 ± 0.007	[0.508, 0.54]	3
J4	3 d	0.544 ± 0.007	[0.527, 0.561]	3
J4	7 d	0.564 ± 0.003	[0.557, 0.570]	3
J4	28 d	0.584 ± 0.007	[0.566, 0.601]	3

**Table 5:** Calibration parameters.

		Macroscopic Parameters	Microscopic Parameters						
			Particle Parameters				PB Model		
		*E/* *GPa*	R_1_/ mm	*Ρ/* kg·m^−3^	*E_c_/* GPa	tanΦt	E_c_/ GPa	σc/ MPa	σ_b_ MPa
Coarse Aggregate		35.5	10–16	2350	7.2	0.5	3.6	28	2.7
Cement Mortar	Natural Sand	35	0–4	2400	6.8	0.45	3.2	30	2.5
	Coal Gangue Sand	25	0–4	2250	6.0	0.4	2.8	26	1.8

**Table 6:** Simulation schemes.

ID	Specimen Dimensions (Width × Height) /mm	Coal Gangue Sand Mix Ratio	Particle Size Range /mm	Loading Rate /mm·s^−1^
A	100 × 100	25%	3.5~4.75	0.01
B	100 × 100	50%	3.5~4.75	
C	100 × 100	75%	3.5~4.75	
D	100 × 100	100%	3.5~4.75	

## Data Availability

The data presented in this study are available on request from the corresponding author due to the need to protect the privacy and confidentiality of the student participants in this research.
